# Do Depressive Traits and Hostility Predict Age-Related Decline in General Intelligence?

**DOI:** 10.1155/2012/973121

**Published:** 2012-08-29

**Authors:** Erik Lykke Mortensen, John Calvin Barefoot, Kirsten Avlund

**Affiliations:** ^1^Department of Public Health, Section of Environmental Health, Medical Psychology Unit, University of Copenhagen, Øster Farimagsgade 5 A, 1353 Copenhagen K, Denmark; ^2^Center for Healthy Aging, Faculty of Health Science, University of Copenhagen, 2200 Copenhagen N, Denmark; ^3^Institute of Preventive Medicine, Copenhagen University Hospital, Copenhagen, 1353 Copenhagen K, Denmark; ^4^Department of Psychiatry and Behavioral Sciences, Duke University, Medical Center, Durham, NC 27710, USA; ^5^Department of Public Health, Section of Social Medicine, University of Copenhagen, 1353 Copenhagen K, Denmark; ^6^Danish Aging Research Center, Universities of Aarhus, Copenhagen and Southern Denmark, Denmark

## Abstract

Certain personality traits are likely to be associated with stress and distress through the lifespan, and as a consequence these traits may influence the rate of age-related cognitive decline. The present study uses data from the Glostrup 1914 cohort to analyze potential effects of personality on decline in general intelligence over a 30-year period. The Minnesota Multiphasic Personality Inventory was administered at a 50-year baseline exam, and from this inventory the Obvious Depression Scale and an abbreviated version of the Cook-Medley Hostility Scale were derived. At the 50-year baseline and at the 60-, 70-, and 80-year followups the full version of Wechsler's Adult Intelligence Scale (WAIS) was administered to 673, 513, 136, and 184 participants. Mixed effects statistical models were used to evaluate both the effect of the personality scores on level of intelligence and the interaction between the personality scores and the time since followup. Analyses were adjusted for demographic background and a wide range of lifestyle factors. Both obvious depression and hostility were negatively associated with level of intelligence, but personality scores did not influence rate of decline in general intelligence.

## 1. Introduction

Recent decades have seen a widespread interest in personality and health [1]. This interest partly reflects an increasing focus on personality in a lifespan perspective and on personality and aging. Although personality, aging, and well-being have been investigated in several studies [2], there is very little research on the associations between personality and age-related changes in cognition. This is remarkable because much evidence suggests that stress may impact brain structures involved in cognition [3], and that personality traits associated with increased vulnerability to stress may influence cognition over the lifespan [4]. Thus, Crowe et al. [4] suggested that personality traits associated with stress, depression, and anxiety may be associated with age-related cognitive decline and risk of Alzheimer's disease and demonstrated that the broad personality dimension of neuroticism was in fact associated with higher risk of cognitive impairment. The study was limited by lack of early-life assessment of cognition and by the use of a relatively rough binary measure of cognition based on a telephone interview. However, neuroticism is known to be associated with increased reactivity to environmental stress [5] and has been found to be associated with psychopathology [6], including depression, which is a relatively well-documented risk factor for dementia [7]. If associations between neuroticism and normal age-related cognitive decline could be demonstrated over a wide range of the lifespan, the public health implications would be substantial since normal cognitive decline as a consequence of personality-related vulnerability to stress would affect a much larger part of the general population and presumably would affect individuals over a larger part of their life-span.

Obviously, risk factors for Alzheimer's disease or other forms of dementia are not necessarily risk factors for normal age-related decline. However, studies of personality and normal age-related cognitive decline involve a number of methodological challenges. Thus, cross-sectional studies of old age samples are unable to separate effects of personality on age-related cognitive decline from the associations between personality and cognitive performance which can be demonstrated in younger adults [8]. Studies with short followup intervals will usually observe relatively small age-related changes and may consequently not have sufficient power to detect associations between personality and cognitive decline.

In this perspective, the Glostrup 1914 cohort offers an unusual opportunity to analyze potential associations between personality and cognitive decline as the Minnesota Multiphasic Personality Inventory (MMPI) was administered at age 50 and the cohort was repeatedly assessed with the same cognitive tests for up to 45 years [9]. Among the many scales which can be derived from the MMPI, the Obvious Depression Scale and a short version of the Cook-Medley Hostility Scale have previously been analyzed as predictors of morbidity and mortality [10, 11]. Both scales have been shown to predict cardiovascular disease in the 1914 cohort, and there is a large literature on associations between cardiovascular factors and cognitive decline [12]. Consequently, associations between the two scales and age-related cognitive decline may be hypothesized.

Several findings in nonclinical samples suggest that the Obvious Depression Scale primarily reflects stable aspects of neuroticism and to a smaller extent symptoms of depressive states. In the 1914 cohort 10- and 30-year retest correlations were 0.71 and 0.48 for the Obvious Depression Scale [13], and in another Danish sample of 584 healthy twins, the Obvious Depression Scale was demonstrated to correlate 0.69 with the Neuroticism Scale of the NEO-PI-R [14]. Trait anxiety and trait depression are core components of neuroticism as a broad dimension of personality, and consequently effects on cognitive decline of state anxiety and state depression must be separated from effects of personality. In a cross-sectional study the NEO personality factors explained up to 7% of the variance in cognition, while the state anxiety and state depression scales of the State-Trait Personality Inventory explained very little additional variance [15]. However, a study including a 3-year followup observed no associations between neuroticism and cognitive performance [16].

Hostility may be associated with a tendency to perceive the social environment as stressful and to elicit antagonistic reactions from other people [17]. However, few studies have investigated associations between hostility and age-related cognitive decline. A study using an abbreviated version of the Cook-Medley Hostility Scale found that hostility, in particular hostile attributions, predicted cognitive decline [18]. A more recent study of a community sample used 8 cynicism items from the Cook-Medley Hostility Scale and found that hostility was associated with baseline level of cognitive functioning, but did not influence decline over a 6-year period [19]. However, it is still possible that hostility would influence decline over a longer followup period, and the main aim of the present study was to investigate the obvious depression and Cook-Medley hostility scores at age 50 as predictors of cognitive decline over the following three decades of the lifespan. 

Many previous studies have focused on tests of specific cognitive functions, such as reaction time [20], perceptual speed [21], and memory [22]. Studies of the Glostrup 1914 cohort incorporated the full Wechsler Adult Intelligence Scale (WAIS) and consequently, the data provide a unique opportunity to evaluate potential associations between personality and age-related decline in general intelligence. Previous analysis of the 1914 cohort has shown substantially more decline for the performance than for the Verbal IQ [23, 24], and consequently we expected the Performance IQ to be more sensitive to any effects that personality may have on age-related cognitive decline.

Measures of intelligence are usually strongly associated with demographic factors such as education and social status and to some extent this may also be the case for personality measures of depressive traits and hostility. Both intelligence and personality traits may also be associated with lifestyle factors [25], and consequently any observed association between personality and decline in intelligence may reflect confounding by demographic and lifestyle factors. Fortunately, the available data for the 1914 cohort made it possible to control for a range of these potentially confounding factors.

## 2. Materials and Methods

### 2.1. Participants

In 1964, the Copenhagen County Hospital and the County Mental Hospital initiated a population study of all people born in 1914 and living in a predefined administrative area close to the two hospitals in Glostrup. A total of 976 individuals were eligible for the study, and of these 802 50-year olds participated in the medical and 698 in the psychological part of the study. Follow-up studies were conducted during the next 45 years, the most recent being a 95-year followup. 

In 1964 673 of the 698 participants (384 men and 289 women) completed both the WAIS and the Danish version of the MMPI. The present study sample comprises these 673 individuals and the subsamples participating in the 60-, 70- and 80-year followups conducted in 1974 (*n* = 513 : 293 men and 220 women), 1984 (*n* = 136 : 71 men and 65 women), and 1995 (*n* = 184 : 91 men and 93 women). Details about the medical [26] and psychological studies [9] of the cohort are provided elsewhere.

### 2.2. Measures

#### 2.2.1. Cognitive Assessment

In the 50-, 60-, and 80-year studies the complete WAIS [27] was administered to all participants. When the 50-year-baseline study of the Glostrup 1914 cohort was initiated in 1964, the original version of the WAIS had just been translated into Danish. It consists of six verbal subtests (information, comprehension, similarities, arithmetic, digit span, vocabulary) and five performance subtests (digit-symbol, picture completion, block design, picture arrangement, and object assembly). Testing procedures and scoring criteria have previously been described in detail [28, 29]. To permit comparison between successive WAIS followups, all IQs are based on the Danish 50-year norms [30]. Only one tester was available for the 70-year followup, and consequently the complete WAIS was only administered to 141 participants, of whom 136 had completed the MMPI and were included in the present study sample. At the 80-year followup 329 of the 698 50-year participants were still alive, and of these 189 participated in the followup (184 had completed the MMPI and are included in the present study sample). As described previously, the 189 participants in the 80-year study obtained higher mean full-scale IQ at the 50-year baseline than the remaining participants in the 50-year study [23]. 

#### 2.2.2. Obvious Depression Scale

 The Danish version of the MMPI [31] consists of 408 true-false items, although “don't know” is a third answer category in the Danish version. The Obvious Depression Scale comprises 40 items: 8 items potentially reflecting physical health and 32 items reflecting mood, feelings of well-being, and self-esteem [11]. Coefficient alpha for the Danish 50-year sample was 0.78 for the Obvious Depression Scale. 

#### 2.2.3. Cook-Medley Hostility Scale

The Danish version of the MMPI did not include all the 566 items of the complete inventory. Consequently, the present study included only 27 of the 39-item abbreviated Cook-Medley Hostility Scale: 11 items on cynicism, 9 on hostile attributions, 4 on aggressive responding, and 3 items on hostile affect [32]. Coefficient alpha for the Danish 50-year sample was 0.82 for the 27 item Hostility Scale.

#### 2.2.4. Demographic Covariates

These included sex, education, and social status assessed at the 50-year study. School education was coded on a 3-point scale (primary to upper secondary) and vocational training on a 5-point scale (no vocational training to academic). The two scores were combined into an overall ordinal index of educational level with a 2–8 point range [23]. Social status was ranked in 6 ordinal levels based on educational level, job position, and number of subordinates [33].

#### 2.2.5. Lifestyle Factors

These included measurement of systolic blood pressure, smoking (as a binary variable), physical activity at work and at leisure (both four-level ordinal measures, recoded into three categories) [11], obesity (measured by body mass index (BMI)), and measures of total serum cholesterol, triglycerides, and insulin [26] analyzed in blood samples obtained in the morning after a 13-hour fast.

### 2.3. Data Analysis

Mixed-effects models were used to test associations between the two personality traits and level of WAIS performance and to test associations between personality traits and change in cognitive function. Data were analyzed using the xtmixed procedure of Stata version 12 (StataCorp LP, College Station, TX, USA). At the 50-year baseline, information on BMI, blood pressure, total serum cholesterol, triglycerides, and insulin was missing for 2, 13, 27, 29, and 27 individuals, respectively. To avoid diluting the sample in multivariate analyses including these variables, the relatively few missing values were imputed using the multivariate normal regression imputing facilities of Stata.

A basic growth curve model, including time since the 50-year baseline as both a fixed and random effect and assuming both random intercept and slope, was used in preliminary analyses of the WAIS full-scale IQ. Since decline in WAIS IQs was nonlinear, time squared was included to allow for non-linear change. Sex, education, and social status were considered core confounders explaining substantial variance in full-scale IQ, and these variables were included in a series of models, which for each continuous covariate tested linear and quadratic regression, and for all covariates tested the significance of the interaction with the time since baseline variable. None of the interactions with the time variable was significant, and significant quadratic effects were only observed for social status and insulin level. Consequently, mean-centered squared variables were included in models with these two variables.

For the obvious depression and hostility scales both main effects and interactions with the time variable were tested in the following models: Model 1 was the basic model including time since the baseline (and the squared time variable). Model 2 also included the sociodemographic variables of sex, education, and social status, including the centered squared status variable. Finally, model 3 further included the lifestyle variables of systolic blood pressure, serum cholesterol, triglycerides, insulin (including a quadratic term), BMI, work and leisure physical activity, and smoking. All three models were analyzed with the full-scale, verbal, and Performance IQs as outcome and with time since the 50-year baseline as both a fixed and random effect and assuming both random intercept and slope, corresponding to assuming random variation in level and form of the individual growth curves.

There was some evidence of selective attrition, and consequently the main analyses were repeated on a reduced longitudinal sample comprising the 184 individuals who participated in both the 50-year and 80-year studies. 

The growth curve models provide random effect estimates of interindividual variance in level and slope of the decline curves. By comparing variance estimates for models with and without the obvious depression or the Cook-Medley Hostility Scale, it is possible to calculate the reduction in variance associated with including each of the MMPI scales in the models. 

All statistical tests were two-sided and determined significant at the 5% level.

## 3. Results

The sample characteristics for the 50-year baseline and the three followups are shown in Table 1. The percentage of men and smokers decreased at the later followups, and the mean systolic blood pressure and triglycerides level fell. Notably, the mean educational level was higher at the 80-year followup, while the mean social status was lower. For all these variables, significant differences were observed when the 50-year baseline values of the 80-year sample was compared with the baseline values of the remaining 50-year sample. This difference was not significant for any of the remaining variables in Table 1.

Table 2 presents means and standard deviations for the two MMPI scales at the 50-year baseline. For both scales most of the possible score range was used. The distributions were reasonably symmetric with a positive skewness of 0.80 for the Obvious Depression Scale and 0.22 for the abbreviated Cook-Medley Hostility Scale. Women scored significantly higher than men on the Obvious Depression Scale and significantly lower than men on the Cook-Medley Hostility Scales.

Table 3 displays the observed mean IQs at baseline and the three full followup samples as well as means for the subsample comprising the 184 individuals who participated in both the 50- and 80-year studies. Table 3 also presents the mean difference between the results of the 50-year baseline and the 80-year followup. The relatively large standard deviations of this difference illustrate the substantial individual differences in decline which have previously been described [23], and which were further confirmed by the variance in slope (decline in intelligence) observed in all growth-curve models in the present study. Table 3 also shows the high retest correlations which should be compared with the 20-year retest correlations reported previously (for the full-scale IQ, the retest correlations were 0.94 from 50 to 60 years and 0.90 from 50 to 70 years) [9, 24]. 

For all three WAIS IQs, little decline was observed from age 50 to age 60, but decline was obviously increasing and was substantial from age 70 to age 80, particularly for the Performance IQ. Thus, the means in the table indicate that any model describing cognitive development during the 30-year followup period should include a quadratic trend.

**Figure 1:**
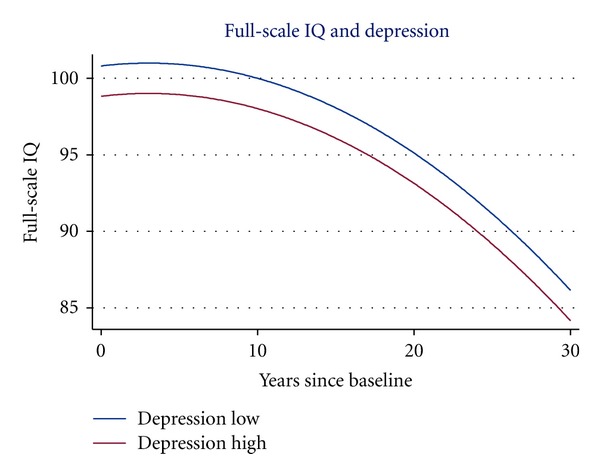
Obvious depression and decline in full-scale IQ from age 50 to age 80. The curves correspond to 1 SD below and above the mean on the Obvious Depression Scale. Adjusted for sex, education and social status.

Table 4 presents the analyses of the Obvious Depression Scale. The table shows coefficients for the scale in models only including a term for the main effect and in models adding a term for the interaction between the scale and the time since baseline variable. The table shows relatively large effects of obvious depression on all three IQs in the unadjusted models, corresponding to a 5-6 percent reduction in the estimated variance of the curve level. However, the effects became dramatically smaller (and nonsignificant for Verbal IQ) when adjusted for demographic background variables (1 percent or less reduction in curve level variance), while adjustment for the set of lifestyle factors hardly changed the estimates. None of the tests of interaction with the time since baseline variable was approaching significance, and these models accordingly showed negligible reduction in the estimates of slope variance. Thus, it must be concluded that obvious depression did not influence the form of the cognitive decline curve, while it was associated with the level of the curve (see Figure 1). 

**Figure 2:**
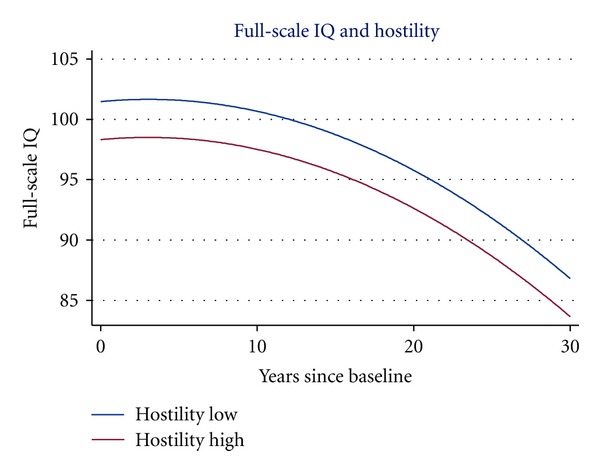
Hostility and decline in full-scale IQ age 50 to age 80. The curves correspond to 1 SD below and above the mean on the abbreviated Cook-Medley Hostility Scale. Adjusted for sex, education, and social status.

Table 5 presents the analyses of the Cook-Medley Hostility Scale. Compared with obvious depression smaller effects of Hostility on all three IQs were observed in the unadjusted models, corresponding to about 3 percent reduction in the estimated variance of the curve level. Although the coefficients became smaller when adjusted for demographic background variables, they remained significant for all three IQs both in models without and with adjustment for the lifestyle factors. For these models, the inclusion of the Cook-Medley Hostility Scale was associated with about 2 percent reduction in curve level variance. None of the tests of interaction with the time since baseline variable was approaching significance, and these models showed negligible reduction in the estimates of slope variance. Thus, scores on the Cook-Medley Hostility Scale were associated with the level of the cognitive performance, but did not influence the form of the decline curve (see Figure 2). 

The models in Tables 4 and 5 only tested the linear effects of obvious depression and hostility. However, models including a quadratic term and testing both its main effect and interaction with time since baseline found no significant quadratic effects of either scale. Correlation coefficients can be used to express the size of the linear associations between the MMPI scales and intelligence. In the 50-year-baseline sample the bivariate correlations of the Obvious Depression Scale were −0.24, −0.22, and −0.24 with the full-scale, the verbal and the Performance IQs, but when adjusted for demographic variables, the corresponding partial correlations were only −0.09, −0.05 and −0.10. For the Cook-Medley Hostility Scale, the correlations were −0.19, −0.17, and −0.17, and the adjusted partial correlations were −0.15, −0.13, and −0.13, confirming a stronger adjusted association with WAIS performance for this scale compared with the Obvious Depression Scale. 

The correlation between the Obvious Depression Scale and the Cook-Medley Hostility Scale was 0.30. When both MMPI scales were included in the same model, only the main effect of the Cook-Medley Hostility Scale remained significant in all models. Thus, the regression coefficients for the two scales were −0.10 (*P* = 0.25) and −0.27 (*P* = 0.003) in the fully adjusted model while the regression coefficients were −0.19 (*P* = 0.023) and −0.31 (*P* < 0.001) in the corresponding models including either the Obvious Depression Scale or the Cook-Medley Hostility Scale.

Since significant sex differences were observed on both scales, it is possible that the influence of obvious depression and hostility is different in men and women. If this were the case, the three-factor interaction between sex, time since baseline, and each of the two personality scales should be significant. However, this interaction was not found to be significant in supplementary analyses adjusting for demographic variables and also analyses adjusting for lifestyle factors.

The supplementary analyses based on the reduced longitudinal sample showed fewer significant associations between the MMPI scales and intelligence level, but otherwise showed essentially the same results as the analyses of the full sample.

## 4. Discussion

The present study based on multiple administrations of the full WAIS and a 30-year followup period showed that the personality traits obvious depression and hostility were associated with the level of WAIS performance, but did not influence the rate of decline in general intelligence during the 30-year followup period. The association with level of WAIS performance to some extent reflected confounding by demographic factors such as sex, education, and social status, but, except for Verbal IQ, the effects remained significant when these covariates were included in the statistical models. The effect estimates hardly changed when the models also included a wide range of lifestyle related variables.

While the low, but significant, negative correlation between obvious depression and intelligence is consistent with the relatively well-documented negative association between neuroticism and intelligence [8, 34], to our knowledge few—if any—studies exist on the relationship between personality and decline in general intelligence. Many studies have investigated depressive symptoms as a risk factor for dementia [7], while fewer studies have investigated associations between depressive symptoms and cognitive decline [35, 36]. A study based on a large sample and comprehensive assessment of cognitive functions observed no association [37], while another large-sample study observed an association between average depressive symptom score and cognitive decline over varying followup intervals with a mean length of 4.4 years [38]. Among the available studies, the present study is unique because of the young age of the participants at baseline, the long followup period, and the instruments used to assess depression and cognitive function: The participants were only 50 years old at baseline, the followup interval was exceptionally long, and our study used general intelligence as outcome. It is true that many studies have evaluated associations between depression and cognition, but these studies have typically assessed symptoms of depression within the last week in much older individuals, and they have typically focused on depression as a risk factor for dementia over much shorter followup intervals.

Personality traits reflect stable characteristics of an individual and the distinction between state and trait can only be made by empirical studies of stability over time. The high long-term stability of the obvious depression score described in the introduction suggests that this scale should primarily be considered a measure of depressive traits, but in addition there are important differences between the Obvious Depression Scale and scales assessing acute depressive states: typical measures of depressive symptomatology such as the SCL-90-R [39] and the CES-D [40] ask the respondent about symptoms within the last week, while the MMPI does not specify any time period, but asks the respondent to indicate whether the item is characteristic of the respondent which—in the context of items asking about self-confidence, being tense at work, liking parties, and about being happy most of time—is likely to be interpreted by the respondent as questions about his or her personality. Thus, the instruction to the respondent and the item content differentiate trait and state measures, and in the case of the Obvious Depression Scale make the high correlation with the Neuroticism Scale of the NEO-PI-R understandable [14].

For hostility, a recent study observed a measure of cynical hostility to be associated with lower cognitive function, but not with decline in cognition [19]. We observed essentially the same results with a longer followup period and with a measure of general intelligence as cognitive outcome. 

Generally, few predictors of cognitive decline have been identified based on strong evidence [12] which may partly reflect complex methodological problems. In spite of the comprehensive assessment of intelligence, and in spite of the long followup period sample attrition is an obvious limitation of our study since, for each outcome, the study was based on 1506 observations, of which only 136 and 184 were from the 70- and 80-year followups. Thus, statistical power may not have been sufficient to detect weak associations between personality and decline in intelligence, particularly since cognitive change was only substantial at the last two followups. 

If the negative findings with respect to influence of personality on the rate of cognitive decline reflect a statistical power problem, stronger effects may be expected on the outcome showing the most substantial decline. Over the 30-year followup period Table 3 shows about 1 standard deviation decline for Performance IQ, but only about half a standard deviation for Verbal IQ. Thus, clearer evidence of effects on decline was anticipated for the Performance IQ, but Tables 4 and 5 show no significant interactions for the Performance IQ. Furthermore, separate analyses of each of the WAIS subtests as outcome also showed no significant interactions between the MMPI scales and time since baseline (data not shown). However, potential effects of personality on age-related decline in specific cognitive functions should be further investigated.

The MMPI scales were not available for all followups, which is one of the reasons that we decided to conduct a prediction study based on the MMPI and covariate data from the 50-year baseline study. However, this may be considered a weakness of the study, to the extent that the traits assessed by the two MMPI scales may change during the long followup interval and to the extent that the 50-year scores were influenced by variance in the mental state of the participants. In particular, scores on the Obvious Depression Scale may reflect both trait and state components, although high retest correlations have previously been reported for this scale [13]. We observed substantial ten-year retest correlations from the 50-year baseline to the 60-year followup: 0.74 for the Cook-Medley Hostility Scale and 0.67 for the Obvious Depression Scale, and lack of stability is unlikely to be a major problem since the scales have previously been demonstrated to predict morbidity and mortality over long followup periods [10, 11]. 

Interpretations of the associations between low intelligence and traits such as obvious depression and hostility are ambiguous because they do not necessarily reflect effects of personality on intelligence or cognitive development since reverse causation—that is, effects of intelligence on personality or personality development—is also a possibility. Indeed, the associations need not reflect a causal relationship since both cognitive development and personality development may be related to factors such as adverse social conditions in childhood [41]. We were unable to control childhood history and social circumstances, but we did adjust for educational level and social status at the 50-year baseline. Tables 4 and 5 clearly show that a substantial part of the covariance between the MMPI scales and WAIS performance reflects association with education and social status. However, intelligence is likely to be one of the factors influencing achieved educational level, and therefore controlling for education may eliminate part of the variance in the association between personality and intelligence that is not due to confounding. In contrast, the tables show remarkably small effects of including a wide range of lifestyle-related factors in the statistical models. As discussed elsewhere, it has proven difficult to identify consistent effects of lifestyle factors on the rate of cognitive decline in the 1914 cohort [9], which is in line with the relatively small effects observed in major longitudinal cohort studies [42, 43].

To the extent that depression and hostility reflect stable personality characteristics, they are likely to be associated with stress and distress across the lifespan. This is the main rationale for the hypothesis that these personality traits influence cognitive decline since increased levels of stress may either influence brain functioning and cognition directly or indirectly through an association with cardiovascular dysfunction or disease [4, 19]. If effects of personality on decline in general intelligence cannot be demonstrated, this may be because the brain is less vulnerable to high levels of stress than expected, or because personality is only one out of a multitude of determinants of cognitive decline. Future studies should focus on the interaction between personality and other determinants of cognitive decline and track changes in personality and cognition across the lifespan to illuminate the direction of causality. 

## Figures and Tables

**Table 1:** Sample characteristics at 50-year baseline and the three followups^1^.

Variable	50-year baseline	60-year followup	70-year followup	80-year followup
Number of participants	673	513	136	184
Demographic variables				
Men (%, *n*)	57 (384)	57 (293)	52 (71)	49 (91)*
Education (mean, SD)	3.6 (1.4)	3.6 (1.4)	3.6 (1.3)	3.8 (1.4)*
Social status (mean, SD)	4.5 (1.1)	4.5 (1.1)	4.5 (0.9)	4.3 (1.0)*
Lifestyle factors				
Smokers (%, *n*)	69 (461)	67 (339)*	66 (90)	55 (101)*
Sedentary work activity (%, *n*)^2^	27 (183)	27 (139)	24 (32)	32 (58)
Sedentary leisure activity (%, *n*)	18 (124)	17 (89)	15 (20)	17 (31)
BMI (mean, SD)	25.2 (4.0)	25.2 (3.8)	25.4 (4.1)	25.0 (3.3)
Systolic BP (mean mmHG, SD)	138.7 (20.4)	137.9 (19.4)	134.9 (18.2)*	135.2 (18.0)*
Total cholesterol (mean mg/dL, SD)	285.7 (49.5)	285.6 (49.9)	286.0 (47.9)	288.9 (47.7)
Fasting insulin (mean units/mL, SD)	22.6 (7.1)	22.5 (7.2)	23.4 (8.6)	22.7 (7.8)
Triglycerides (mean mM/liter, SD)	111.2 (68.4)	110.0 (70.5)	113.0 (76.1)	101.6 (50.9)*

^1^An* indicates that there was a significant difference on the variable between the followup subsample and the remaining part of the 50-year baseline sample.

^2^This percentage includes about 11% of the participants who reported no work.

**Table 2:** Score distributions on obvious depression and Cook-Medley Hostility Scales at age 50.

MMPI scale	*N*	Mean	SD	Range
Obvious depression scale^1^	673	11.48	5.38	1–34
Men	384	10.21	4.99	1–34
Women	289	13.18	5.42	4–30
Cook-Medley hostility scale^2^	673	10.43	4.92	0–25
Men	384	10.97	5.04	1–23
Women	289	9.72	4.67	0–25

^1^Women scored significantly higher than men (*P* < 0.001).

^2^Men scored significantly higher than women (*P* = 0.001).

**Table 3:** Mean WAIS IQs during the lifespan from 50 to 80.

Followup	*N*	Full scale IQ		Verbal IQ		Performance IQ	
Full samples		Mean	SD	Mean	SD	Mean	SD
50-year baseline	673	98.96	14.40	98.19	14.21	99.12	14.22
60-year followup	513	98.84	14.33	99.21	14.59	97.65	13.80
70-year followup	136	94.90	14.12	96.22	13.71	93.40	14.37
80-year followup	184	87.66	15.49	92.27	15.64	83.46	14.78
*80-year sample* ^ 1^	184						
50-year baseline		102.10	13.79	100.85	13.84	102.31	13.28
80-year followup		87.66	15.49	92.27	15.63	83.46	14.78
Difference		14.44	8.88	8.58	8.75	18.85	10.43
Retest correlation		0.82		0.83		0.73	

^1^Data are for the 184 participants who completed the WAIS at both the 50- and 80-year studies.

**Table 4:** Obvious depression scale: effects on WAIS IQs from 50 to 80.

Model	Coefficient^1^	95% CI	*P* for main effect	Interaction^2^ coefficient	*P* for interaction
Full-scale IQ					
Model 1: unadjusted^3^	−0.639	(−0.83)–(−0.44)	<0.001	0.003	0.319
Model 2: demographics^4^	−0.184	(−0.35)–(−0.02)	0.029	0.003	0.291
Model 3: lifestyle factors^5^	− 0.190	(−0.36)–(−0.03)	0.023	0.003	0.294
Verbal IQ					
Model 1: unadjusted^3^	− 0.572	(−0.77)–(−0.38)	<0.001	0.002	0.638
Model 2: demographics^4^	− 0.119	(−0.28)–(0.05)	0.156	0.002	0.613
Model 3: lifestyle factors^5^	− 0.125	(−0.29)–(0.04)	0.131	0.002	0.616
Performance IQ					
Model 1: unadjusted^3^	− 0.595	(−0.78)–(−0.41)	<0.001	0.005	0.227
Model 2: demographics^4^	− 0.223	(−0.40)–(−0.05)	0.012	0.005	0.236
Model 3: lifestyle factors^5^	− 0.228	(−0.40)–(−0.06)	0.009	0.005	0.236

^1^Fixed effect coefficient from a model only including the main effect of the depression scale. See Section 3 for description of the effects of obvious depression on the estimates of the random effects.

^2^Interaction coefficient from a model including a term for interaction between the obvious depression scale and time since baseline. Additionally all relevant main effects are included in the model.

^3^Model includes linear and quadratic time since baseline and the obvious depression scale.

^4^Model additionally includes sex, education, and social status.

^5^Model additionally includes sex, education, social status, systolic blood pressure, smoking, BMI, total cholesterol, triglycerides, insulin, and leisure and work physical activity.

**Table 5:** Abbreviated Cook-Medley hostility scale: effects on WAIS IQs from 50 to 80.

Model	Coefficient^1^	95% CI	*P* for main effect	Interaction^2^ coefficient	*P* for interaction
Full Scale IQ					
Model 1: Unadjusted^3^	− 0.524	(−0.74)–(−0.31)	<0.001	0.003	0.378
Model 2: Demographics^4^	− 0.320	(−0.49)–(−0.15)	<0.001	0.003	0.386
Model 3: Lifestyle factors^5^	− 0.311	(−0.48)–(−0.14)	<0.001	0.003	0.391
Verbal IQ					
Model 1: Unadjusted^3^	− 0.496	(−0.71)–(−0.28)	<0.001	0.002	0.655
Model 2: Demographics^4^	− 0.283	(−0.45)–(−0.11)	0.001	0.001	0.690
Model 3: Lifestyle factors^5^	− 0.266	(−0.44)–(−0.10)	0.002	0.001	0.699
Performance IQ					
Model 1: Unadjusted^3^	− 0.451	(−0.66)–(−0.24)	<0.001	0.005	0.269
Model 2: Demographics^4^	− 0.295	(−0.48)–(−0.11)	0.002	0.005	0.272
Model 3: Lifestyle factors^5^	− 0.297	(−0.48)–(−0.12)	0.001	0.005	0.280

^1^Fixed effect coefficient from a model only including the main effect of the hostility scale. See Section 3 for description of the effects of hostility on the estimates of the random-effects.

^2^Interaction coefficient from a model including a term for interaction between the abbreviated Cook-Medley Hostility Scale and time since baseline. Additionally all relevant main effects are included in the model.

^3^Model includes linear and quadratic time since baseline and the abbreviated Cook-Medley hostility scale.

^4^Model additionally includes sex, education and social status.

^5^Model additionally includes sex, education, social status, systolic blood pressure, smoking, BMI, total cholesterol, triglycerides, insulin and leisure and work physical activity.
